# *ESR1* and *PIK3CA* Polymorphisms as Potential Genetic Susceptibility Markers for Breast Cancer Risk in Bangladeshi Women: A Case‐Control Study

**DOI:** 10.1155/humu/3651302

**Published:** 2026-07-30

**Authors:** Mahim Hassan Chowdhury, Faria Billal Shaolin, Nishat Tabassum Khusbu, Upama Gope Puja, Md. Sharif Hossain, Md. Robiul Islam, Akash Majumder, Shah Musallin Hassan, Mohiuddin Ahmed Bhuiyan, Md. Aminul Haque

**Affiliations:** ^1^ Research Lab, Rufaida BioMeds, Dhaka, Bangladesh; ^2^ Department of Pharmacy, University of Asia Pacific, Dhaka, Bangladesh, uap-bd.edu; ^3^ School of Pharmacy, BRAC University, Dhaka, Bangladesh, bracu.ac.bd

**Keywords:** breast cancer, cancer risk, *ESR1* gene, genetic marker, *PIK3CA* gene, SNP

## Abstract

Breast cancer (BC) is one of the most common and deadly cancers affecting women worldwide. This study is aimed at investigating the association between BC risk and two single nucleotide polymorphisms (SNPs): *ESR1* (rs2234693) and *PIK3CA* (rs6443624) in a Bangladeshi population. A case‐control study was conducted with 112 BC patients and 124 healthy controls (HCs). Genomic DNA was extracted from peripheral blood samples, and genotyping was performed using polymerase chain reaction‐restriction fragment length polymorphism (PCR‐RFLP). Genotype and allele frequencies were analyzed to assess their association with BC risk. Genotype distributions for both *ESR1* and *PIK3CA* conformed to Hardy–Weinberg equilibrium. The CT genotype of *ESR1* was associated with a reduced risk of BC, whereas the CA genotype of *PIK3CA* was linked to an increased risk. The dominant model for *ESR1* (CT + TT vs. CC) demonstrated a significant protective effect (aOR = 0.288, 95% CI: 0.160–0.516), whereas the dominant model for *PIK3CA* (CA + CC vs. AA) showed a higher risk (aOR = 4.166, 95% CI: 2.363–7.347). Over‐dominant models supported these findings, while recessive models for both SNPs showed no significant associations. The findings suggest that *ESR1 (*rs2234693) may have a protective role and *PIK3CA* (rs6443624) may increase susceptibility to BC in Bangladeshi women. These SNPs may provide preliminary evidence for potential use as genetic susceptibility markers, but larger multicenter studies are needed before any clinical application can be considered.

**Highlights**

• The *PIK3CA* CA genotype is linked to a higher risk of BC, especially in the dominant (aOR = 4.166) and additive (aOR = 4.767) models.
• No significant associations were found for either *ESR1* or *PIK3CA* in the recessive models.
• *ESR1* (rs2234693) acts as a protective factor, while *PIK3CA* (rs6443624) contributes to increased BC susceptibility.

## 1. Introduction

Cancer remains a major global health challenge, ranking as the second leading cause of death in many countries [1]. Breast, lung, prostate, and colorectal cancers account for over 50% of all cancer‐related deaths. In the United States, approximately 2 million people are expected to be diagnosed with cancer by 2024, including 310,720 women and 2790 men with BC [2]. In 2022, BC affected 2.3 million women worldwide, causing 670,000 deaths [3]. In Bangladesh, breast cancer is the leading cancer among women, accounting for 12,989 new cases and 6,162 deaths in 2022, underscoring its substantial and growing public health burden [4].

BC is a complex disease influenced by a multitude of internal and environmental factors, among which genetic predisposition plays a pivotal role. Genetic polymorphism refers to the alteration in DNA sequence leading to phenotypic variation and often to susceptibility due to impacts on gene expression and functionality which are heritable [5]. Single nucleotide polymorphisms (SNPs) are among the most widespread forms of genetic variations inside the human genome. SNPs regulating the life cycle of cells, DNA mismatch repair maintenance, metabolic processes, and immunity have been suggested to elevate the risk to certain cancers [6]. Even though the etiology of BC is complicated, many studies indicate that some SNPs provide a major function in developing BC [7].

The *ESR1* gene, located at chromosome 6q25.1, encodes the estrogen receptor alpha (ER*α*), a nuclear receptor that upon binding estrogen, acts as a transcription factor to regulate genes involved in cell proliferation, differentiation, and other estrogen‐mediated processes [8]. Once estrogen binds to ER*α*, it undergoes dimerization, binds estrogen response elements, or interacts with other transcription factors. This activates genes critical for cell cycle progression and antiapoptotic pathways fueling uncontrolled proliferation [9]. Notably, an SNP in Intron 1 of the *ESR1* gene, characterized by a T > C substitution at position −397 bp (rs2234693), has been reported to influence the transcriptional activity of ER*α* [10]. This alteration may subsequently affect estrogen‐mediated signaling pathways, thereby increasing susceptibility to hormone‐dependent cancers such as BC [11, 12]. Different studies have investigated the association between rs2234693 and BC risk, with varying outcomes across different populations [13]. Furthermore, meta‐analyses have consistently shown that the rs2234693 polymorphism is associated with an elevated risk of developing BC, although the significance of other SNPs such as rs2077647, rs2228480, and rs3798577 remains inconclusive [14, 15].

Phosphatidylinositol‐3‐kinase (PI3K), encoded by the *PIK3CA* gene, regulates key intracellular signaling pathways involved in apoptosis resistance, cell transformation, cancer initiation, and proliferation [16]. Activated by growth factors and hormones, dysregulation of *PIK3CA* stimulates AKT, initiating oncogenic cascades that promote uncontrolled cellular growth and tumorigenesis [16, 17]. The PI3K/AKT/mTOR pathway, implicated in cancer for nearly three decades, is now targeted by validated therapeutic agents [18–20]. Somatic mutations in *PIK3CA* are common in various cancers, with up to 40% frequency in primary breast tumors [21]. Located on chromosome 3q26.3, *PIK3CA* spans 20 exons and harbors several polymorphisms, including rs6443624 (C > A) in an intronic region [22–26]. Although studied in multiple cancer types, data on rs6443624 in BC remain limited and inconclusive [26]. Some evidence suggests it may influence transcription, splicing, or expression, whereas others propose its role as an independent prognostic biomarker [26]. Despite population‐based studies, no definitive association between rs6443624 and BC risk has been confirmed, and its role remains under debate [27].

*ESR1* rs2234693 and *PIK3CA* rs6443624 were selected based on previous evidence suggesting their involvement in cancer susceptibility and their biological relevance to pathways implicated in breast carcinogenesis. Furthermore, both variants exhibit common allele frequencies (MAF>5%) in Asian populations according to data from the 1000 Genomes Project and dbSNP databases, supporting their suitability for genetic association studies. In the present study, our aim is to examine the potential association of SNP of the *ESR1* and *PIK3CA* genes with BC in the Bangladeshi population.

## 2. Materials and Methods

### 2.1. Materials

TaKaRa Taq DNA Polymerase Master Mix, restriction enzymes, and DNA ladder were obtained from TAKARA BIO INC. (Japan). Tris‐borate‐EDTA (TBE) buffer was purchased from Solarbio Life Sciences (China), and SeaKem LE Agarose was sourced from Lonza (United States). Genomic DNA Extraction Kit was supplied by Favorgen (United States). Oligonucleotide primers were synthesized by Macrogen (Korea). Midori Green staining dye was obtained from Nippon Genetics (Europe). Additional restriction enzymes were procured from New England Biolabs (NEB, United States).

### 2.2. Study Subjects

The study population consisted predominantly of self‐reported ethnic Bengali women recruited from the National Cancer Research Institute Hospital, Dhaka. Because the study was designed to investigate genetic associations in the general Bangladeshi population rather than among specific ethnic subgroups, detailed ethnicity‐based stratification was not performed. All research participants were free from any significant physical and/or cognitive illness. A thorough screening process including physical examination, neurological assessment, and laboratory testing was conducted to ensure their eligibility

BC patients were eligible for inclusion if they were female patients aged 18 years or older with histopathologically confirmed primary breast cancer and who provided written informed consent. Healthy controls were age‐matched women without a previous history of any malignancy. Individuals were excluded if they had secondary breast cancer, metastatic cancer originating from another primary site, severe systemic illness, or incomplete clinical information. Individuals with a known family history of breast cancer in first‐degree relatives were excluded from the study.

A total of 112 BC patients and 124 HC were included in the study. Diagnosis of BC was carried out by a certified oncologist based on clinical evaluation and histopathological examinations of biopsy specimens in accordance with established institutional and international clinical practice guidelines for BC. Genotyping analyses were performed at Rufaida BioMeds, Bangladesh.

### 2.3. DNA Extraction and Genotyping

Samples of blood were collected from the patients and placed in tubes containing EDTA. Genomic DNA extraction was done with a genomic DNA extraction kit (Favorgen, United States) following the provided protocol. Briefly, the collected blood samples were lysed and then the lysate was transferred into the binding column tubes. Different buffers were used for washing DNA, and nuclease‐free water was used for the elution of DNA, which was stored at −20°C. DNA concentration and purity were assessed using a UV‐Vis spectrophotometer (Shimadzu Corporation, Japan), and samples with an A260/280 ratio between 1.8 and 2.0 were included for downstream analysis.

The PCR (polymerase chain reaction)‐RFLP assay was performed in a thermal cycler (miniPCR, United States) for the identification of SNPs at various genomic locations. An overview of the thermal condition and primer details to amplify the target regions has been included in Table 1. The PCR was conducted with the help of TaKaRa Taq DNA Polymerase Master Mix. The completion of the PCR was confirmed by 1% agarose gel electrophoresis. The PCR products were digested by *PvuII-HF* (at 37°C for overnight) and *AluI* (at 37°C for overnight) restriction enzymes. *PvuII-HF* and *AluI* restriction enzymes examined the SNP at rs2234693 and rs6443624, respectively. Gel electrophoresis with 2% agarose gel stained with midori Green was performed to visualize the digested products. All gels were independently reviewed by two analysts; discrepancies were resolved by repeat amplification and digestion. The different genotypes for *ESR1* rs2234693 were distinguished based on the size of digested fragments such as CC (1374 bp), TT (936 and 438 bp), or CT (1374, 936, and 438 bp). After the digestion for *PIK3CA* rs6443624 by the restriction enzyme, the homozygous CC genotype produced two fragments (262, 93 bp), heterozygous CA genotype produced three fragments (355, 262 and 93 bp) and the AA genotype produced one fragment (355 bp). Details of the primers, thermal cycling conditions, and amplicon sizes used for genotyping *ESR1* rs2234693 and *PIK3CA* rs6443624 are presented in Table 1. To validate the reliability of the data, 20% of the samples were subjected to Sanger sequencing through a commercial service provider, concordance with PCR‐RFLP was 100%.

**Table 1:** Primer sequences, thermal conditions, and amplified products′ size.

Gene, SNP	Primers′ sequence	Thermal condition	Fragments, BP
	F‐forward, R‐reverse		
*ESR1*	F: 5 ^′^‐CTGCCACCCTATCTGTATCTTTTCCTATTCTCC‐3 ^′^	5 min of denaturation at 98°C, then 40 cycles of 98°C for 40 s, 56.4°C for 50 s, and 72°C for 90 s. The final cycle had a 5 min extension at 72°C	1374
rs2234693	R: 5 ^′^‐TCTTTCTCTGCCACCCTGGCGTCGATTATCTGA‐3 ^′^		
*PIK3CA*	F: 5 ^′^‐TAAGATGTGCAGAGTTCGTTGTATG‐3 ^′^	5 min of denaturation at 98°C, then 40 cycles of 98°C for 40 s, 55°C for 40 s, and 72°C for 50 s. The final cycle had a 5 min extension at 72°C	355
rs6443624	R: 5 ^′^‐TTGCCTTTGTAAATATGCTCCATAATC‐3 ^′^		

### 2.4. Statistical Analyses

Statistical analysis was performed using R software (Version 4.4.1; R Foundation for Statistical Computing, Vienna, Austria; https://www.r-project.org/). Hardy–Weinberg equilibrium (HWE) was tested using the HardyWeinberg package in R. Pearson′s chi‐square tests were used to check the association between categorical variables across different genotype groups. Crude odds ratios (ORs) and adjusted odds ratios (aORs) with 95% confidence intervals (CIs) were estimated using univariable and multivariable logistic regression models, respectively. The multivariable models were adjusted for age, body mass index (BMI), menopausal status, education level, socioeconomic status, residence, and occupation.

A post‐hoc statistical power analysis was performed using the statsmodels statistical framework with a two‐sided significance level of 0.05 to evaluate the adequacy of the study sample size. Power calculations were conducted using the observed genotype frequencies under the dominant genetic models at a significance level of *α* = 0.05. The estimated statistical power was 97.8% for *ESR1* rs2234693 and 99.9% for *PIK3CA* rs6443624, indicating sufficient power to detect the observed associations. Model performance was evaluated using McFadden′s pseudo‐R^2^ and the area under the receiver operating characteristic curve (AUC) to assess model fit and discriminatory ability. All statistical tests were two‐sided, and a *p* value < 0.05 was considered statistically significant. Because this study investigated a limited number of prespecified candidate SNPs under complementary genetic inheritance models, no formal correction for multiple testing (e.g., Bonferroni or false discovery rate adjustment) was applied.

### 2.5. Ethical Consideration

The study received approval from the Institutional Review Board (IRB), Brac University (IRB No. BRACUIRB120220005) and all procedures adhered to the principles of the Declaration of Helsinki. All the techniques were conducted with the relevant guidelines and regulations outlined in the approved research protocol. Written informed consent was obtained from all participants prior to their inclusion in the study.

## 3. Results

### 3.1. Association Between BC and *ESR1* rs2234693 Polymorphism

The association between BC and the *ESR1* (rs2234693) polymorphism was evaluated by comparing genotype frequencies between cases (*n* = 112) and controls (*n* = 124) under different genetic models (Table 2). Genotype distributions in both groups adhered to HWE, with *p* values of 0.99 (cases) and 0.786 (controls), confirming the suitability of the data for further analysis.

**Table 2:** Association between BC and *ESR1* rs2234693 polymorphism (multivariable logistic regression adjusted for age, BMI, menopausal status, education level, socioeconomic status, residence, and occupation).

**Allele**	**Cases (** **n** = 112**)**	**HWE**		**Controls (** **n** = 124**)**	**HWE**	
		**X** ^**2**^	**p**		**X** ^**2**^	**p**

CC (common homozygote) (1374 bp)	(*n* = 83; 74.10%)	0.0001	0.99	(*n* = 61; 49.19%)	0.074	0.786
CT (heterozygote) (1374, 936, and 438 bp)	(*n* = 27; 24.10%)			(*n* = 61; 49.19%)		
TT (rare homozygote) (936 and 438 bp)	(*n* = 2; 1.79%)			(*n* = 2; 1.61%)		

**Genetic model**	**Crude model**			**Adjusted model**		
	**OR**	**95% CI**	**p**	**OR**	**95% CI**	**p**

Additive 1 (CT vs. CC)	0.325	0.186–0.570	< 0.001	0.275	0.151–0.501	< 0.001
Additive 2 (TT vs. CC)	0.735	0.101–5.364	0.761	0.979	0.103–9.336	0.985
Dominant (CT + TT vs. CC)	0.338	0.195–0.586	< 0.001	0.288	0.160–0.516	< 0.001
Recessive (TT vs. CC + CT)	1.109	0.154–8.008	0.918	0.993	0.120–8.206	0.995
Over‐dominant (CT vs. CC + TT)	0.328	0.188–0.573	< 0.001	0.278	0.153–0.504	< 0.001

*Note:* Genotype frequencies were tested for Hardy–Weinberg equilibrium (HWE) using the chi‐square test (X^2^). Crude odds ratios (ORs) and adjusted odds ratios (aORs) with 95% confidence intervals (CIs) were estimated using logistic regression models in R. Adjusted models included age, BMI, menopausal status, education level, socioeconomic status, residence, and occupation. All models used the CC genotype (common homozygote) as the reference group. *p* > 0.05 indicates that genotype distribution follows Hardy–Weinberg equilibrium.

Abbreviations: aOR, adjusted odds ratio; CI, confidence interval; HWE, Hardy–Weinberg equilibrium; X^2^, chi‐square value.

The CC genotype (common homozygote) was most frequent, present in 74.1% of cases and 49.2% of controls. The heterozygous CT genotype appeared in 24.1% of cases but was considerably more common among controls (49.2%). The TT genotype was rare, observed in only 1.8% of cases and 1.6% of controls.

Under the additive Model 1 (CT vs. CC), carriers of the CT genotype demonstrated a significantly reduced risk of BC, with an aOR of 0.275 (95% CI: 0.151–0.501; *p* < 0.001). In contrast, additive Model 2 (TT vs. CC) showed no significant association (aOR = 0.979, 95% CI: 0.103–9.336; *p* = 0.985), likely reflecting the very low frequency of TT genotypes.

Similarly, the dominant model (CT + TT vs. CC) confirmed a strong protective effect, with an aOR of 0.288 (95% CI: 0.160–0.516; *p* < 0.001). The recessive model (TT vs. CC + CT) did not show a significant effect (aOR = 0.993, 95% CI: 0.120–8.206; *p* = 0.995). The over‐dominant model (CT vs. CC + TT) again supported the protective role of heterozygosity, with an aOR of 0.278 (95% CI: 0.153–0.504; *p* < 0.001).

Taken together, these findings consistently suggest that individuals carrying the CT genotype of the *ESR1* rs2234693 polymorphism have a significantly lower risk of developing BC. The TT genotype, although present at a very low frequency, did not demonstrate any meaningful association in this study. Larger, population‐based studies will be needed to further clarify the potential contribution of this rare genotype.

### 3.2. Association Between BC and *PIK3CA* rs6443624 Polymorphism

The association between the *PIK3CA* (rs6443624) polymorphism and BC risk was also examined using various genetic models, as presented in Table 3. Genotype distributions in both cases and controls conformed to HWE, with *p* values of 0.598 (cases) and 0.966 (controls), supporting the reliability of the dataset for genetic association analysis.

**Table 3:** Association between BC and *PIK3CA* rs6443624 polymorphism (multivariable logistic regression adjusted for age, BMI, menopausal status, education level, socioeconomic status, residence, and occupation).

**Allele**	**Cases (** **n** = 112**)**	**HWE**		**Controls (** **n** = 124**)**	**HWE**	
		**X** ^**2**^	**p**		**X** ^**2**^	**p**

AA (common homozygote) (355 bp)	(*n* = 32; 28.57%)	0.279	0.598	(*n* = 76; 61.29%)	0.001	0.966
CA (heterozygote) (93, 262, and 355 bp)	(*n* = 79; 70.54%)			(*n* = 41; 33.07%)		
CC (rare homozygote) (93 and 262 bp)	(*n* = 1; 0.89%)			(*n* = 7; 5.65%)		

**Genetic model**	**Crude model**			**Adjusted model**		
	**OR**	**95% CI**	**p**	**OR**	**95% CI**	**p**

Additive 1 (CA vs. AA)	4.576	2.616–8.007	< 0.001	4.767	2.675–8.495	< 0.001
Additive 2 (CC vs. AA)	0.339	0.040–2.871	0.321	0.316	0.033–3.030	0.318
Dominant (CA + CC vs. AA)	3.958	2.292–6.837	< 0.001	4.166	2.363–7.347	< 0.001
Recessive (CC vs. CA + AA)	0.151	0.018–1.244	0.079	0.148	0.017–1.287	0.083
Over‐dominant (CA vs. CC + AA)	4.846	2.790–8.419	< 0.001	4.997	2.827–8.832	< 0.001

*Note:* Genotype frequencies were tested for Hardy–Weinberg equilibrium (HWE) using the chi‐square test (X^2^). Crude odds ratios (ORs) and adjusted odds ratios (aORs) with 95% confidence intervals (CIs) were estimated using logistic regression models in R. Adjusted models included age, BMI, menopausal status, education level, socioeconomic status, residence, and occupation. All models used the AA genotype (common homozygote) as the reference group. *p* > 0.05 indicates that genotype distribution follows Hardy–Weinberg equilibrium.

Abbreviations: aOR, adjusted odds ratio; CI, confidence interval; HWE, Hardy–Weinberg equilibrium; X^2^, chi‐square value.

In the case group, the heterozygous genotype (CA) was predominant, observed in 70.5% of individuals. The common homozygous genotype (AA) was present in 28.6%, whereas the rare homozygous genotype (CC) was detected in only 0.9%. In contrast, among controls, the AA genotype was more prevalent (61.3%), followed by CA (33.1%) and CC (5.6%).

Under additive Model 1 (CA vs. AA), individuals with the CA genotype had a significantly increased risk of BC, with an aOR of 4.767 (95% CI: 2.675–8.495; p <0.001). This strong association suggests that heterozygosity at this locus is a potential risk factor. In additive Model 2 (CC vs. AA), the aOR was 0.316 (95% CI: 0.033–3.030; *p* = 0.318). However, this result was not statistically significant, likely due to the very low frequency of the CC genotype.

The dominant model (CA + CC vs. AA) also revealed a significant association, with an aOR of 4.166 (95% CI: 2.363–7.347; *p* < 0.001), reinforcing the role of the CA genotype in increasing BC susceptibility. In contrast, the recessive model (CC vs. CA + AA) did not yield a significant association (aOR = 0.148; 95% CI: 0.017–1.287; *p* = 0.083), again reflecting the limited presence of the CC genotype.

Lastly, under the over‐dominant model (CA vs. CC + AA), the aOR was 4.997 (95% CI: 2.827–8.832; *p* < 0.001), indicating that individuals carrying the heterozygous CA genotype had an increased risk of breast cancer compared with those carrying either homozygous genotype (AA or CC). This finding further supports the association between the *PIK3CA* rs6443624 polymorphism and breast cancer susceptibility, suggesting that heterozygosity at this locus may contribute to disease risk. In summary, the CA genotype of the *PIK3CA* rs6443624 polymorphism appears to play a significant role in increasing BC risk, particularly under the additive Model 1 and dominant model. The low frequency of the CC genotype limits the interpretive power of the other models, suggesting the need for larger studies to validate these findings.

### 3.3. Comparative Analysis of *ESR1* rs2234693 and *PIK3CA* rs6443624 Polymorphisms

The performance of the multivariable logistic regression models was further evaluated using McFadden′s pseudo‐R^2^ and the AUC. The *ESR1* model demonstrated a McFadden′s pseudo‐R^2^ of 0.071 and an AUC of 0.674, whereas the *PIK3CA* model showed a McFadden′s pseudo‐R^2^ of 0.132 and an AUC of 0.700, indicating acceptable discriminatory performance of both models.

Both *ESR1* rs2234693 and *PIK3CA* rs6443624 polymorphisms exhibited distinct patterns of connection with BC. For *ESR1* rs2234693, the CT genotype demonstrated a significant protective effect across multiple models, suggesting that heterozygosity may confer resistance to BC. In contrast, for *PIK3CA* rs6443624, the CA genotype consistently emerged as a significant risk factor, with strong associations under additive Model 1 and the dominant model.

The very low frequency of the rare homozygous genotypes (TT for *ESR1* rs2234693 and CC for *PIK3CA* rs6443624) highlights the need for larger studies to validate these findings and assess their potential implications. The adherence of genotype distributions to HWE in both polymorphisms strengthens the reliability of these results.

**Figure 1:**
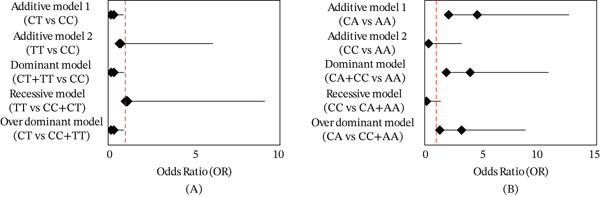
Forest Plot. Association between BC with (A) *ESR1* (rs2234693) polymorphism and (B) *PIK3CA* (rs6443624) polymorphism.

Overall, these findings suggest that the *ESR1* rs2234693 and *PIK3CA* rs6443624 polymorphisms contribute to BC susceptibility in different ways. Although *ESR1* rs2234693 appears to have a protective heterozygous advantage, *PIK3CA* rs6443624 demonstrates a significant risk associated with heterozygosity. Future studies need to focus on further elucidating the underlying biological mechanisms involved and investigating the clinical relevance of these polymorphisms in BC prevention and management. These associations across multiple genetic models are visually illustrated in Figure 1, emphasizing the protective and risk‐enhancing patterns for *ESR1* and *PIK3CA*, respectively.

To investigate the potential influence of age and hormonal status on the observed genetic associations, stratified analyses were performed according to age (≤ 50 and > 50 years) (Table S1) and menopausal status (premenopausal and postmenopausal) (Table S2). The protective association of the *ESR1* rs2234693 dominant genotype was significant among women aged ≤ 50 years and premenopausal women but was not statistically significant among women aged > 50 years or postmenopausal women. In contrast, the *PIK3CA* rs6443624 dominant genotype remained significantly associated with an increased risk of breast cancer across both age groups and menopausal subgroups, although the effect estimates were stronger among younger and premenopausal women.

Associations between the investigated polymorphisms and clinicopathological characteristics were evaluated among breast cancer patients (Table S3). No significant associations were observed between either SNP and ER status, PR status, HER2 status, tumor grade, or tumor size. However, *ESR1* rs2234693 showed a significant association with lymph node involvement (*p* = 0.038), whereas *PIK3CA* rs6443624 was not significantly associated with any clinicopathological characteristic.

### 3.4. Sociodemographic History

The demographic characteristics of BC patients (*n* = 112) and HC (*n* = 124) used in our research are presented in Table 4. The average age of the BC group was 46.9 ± 12.5 years, compared to 47.8 ± 13.1 years in the control group, showing no significant difference (*p* = 0.58). Similarly, the mean BMI did not differ significantly between groups, with values of 25.7 ± 4.9 kg/m^2^ for patients and 25.2 ± 5.1 kg/m^2^ for controls.

**Table 4:** Demographic characteristics of BC and HC.

Variable	Cases (*n* = 112)	Controls (*n* = 124)	*p* ^a^
Age (years)	46.9 ± 12.5	47.8 ± 13.1	0.58
BMI (kg/m^2^)	25.7 ± 4.9	25.2 ± 5.1	0.32
Number of Children	2.13 ± 0.91	2.09 ± 0.87	0.45
Occupation			0.46
Housewife	38 (33.9%)	44 (35.5%)	
Working women	68 (60.7%)	73 (58.9%)	
Student	6 (5.4%)	7 (5.6%)	
Level of education			0.71
Illiterate	60 (53.57%)	60 (48.38%)	
<10 years	40 (35.71%)	48 (38.71%)	
≥10 years	12 (10.71%)	16 (12.9%)	
Socioeconomic status			0.29
Lower class	59 (52.68)	74 (59.68%)	
Middle class	45 (40.19%)	38 (30.66%)	
Higher class	8 (7.14%)	12 (9.68%)	
Residential status			1.0
Rural	76 (67.86%)	85 (68.55%)	
Urban	36 (32.14%)	39 (31.45%)	
Menopausal status			0.881
Premenopausal	49 (43.75%)	52 (41.94%)	
Postmenopausal	63 (56.25%)	72 (58.06%)	

*Note:* Data are shown as mean ± SD (median) for continuous variables and % (n) for categorical variables.

^a^p values: t‐test for continuous variables (age, BMI, number of children); chi‐square test for occupation, level of education, socioeconomic status, residential status, and menopausal status.

Occupational status was also comparable across both groups. Among BC patients, 33.9% were housewives, 60.7% were employed, and 5.4% were students, whereas in the control group, 35.5% were housewives, 58.9% were employed, and 5.6% were students. These differences were not statistically significant (*p* = 0.46). Additionally, the mean number of children was nearly identical; 2.13 ± 0.91 in cases and 2.09 ± 0.87 in controls (*p* = 0.45).

Further sociodemographic analysis revealed that education level (*p* = 0.71), socioeconomic status (*p* = 0.29), residential status (*p* = 1.0), and menopausal status (*p* = 0.881) also showed no significant differences between cases and controls. This indicates that these demographic factors were evenly distributed across both groups and are unlikely to have influenced BC susceptibility in this study population.

### 3.5. Histopathological Characteristics of BC Patients

The clinical and histopathological features of BC cases are summarized in Table 5. All patients presented with a breast lump (100%), whereas 40.19% reported weight loss, 39.29% presented with peau d’orange, and 18.75% experienced nipple discharge. Histological analysis revealed that the majority of tumors were invasive ductal carcinoma (75%), followed by ductal carcinoma in situ (14.29%) and invasive lobular carcinoma (10.71%). Regarding tumor grade, 46.43% of patients were in Grade I, 37.5% in Grade II, and 16.07% in Grade III.

**Table 5:** Histopathological data of BC patients.

Variables	Cases (*n* = 112)
Breast lump	112 (100%)
Nipple discharge	21 (18.75%)
Retraction of nipple	16 (14.29%)
Deviation of nipple	10 (8.92%)
Weight loss	45 (40.19%)
Peau d′orange	44 (39.29%)
Ulceration of overlying skin and/or nipple areola	11 (9.82%)
Histology	
Ductal ca in situ	16 (14.29%)
Invasive lobular	12 (10.71%)
Invasive ductal	84 (75%)
Clinical stages	
Grade I	52 (46.43%)
Grade II	42 (37.5%)
Grade III	18 (16.07%)
Hormone receptor	
ER+	73 (65.19%)
PR+	80 (71.43%)
Her‐2+	32 (28.57%)
Tumor size	
< 2 cm	6 (5.36%)
2.1–5.0 cm	62 (55.36%)
> 5.0 cm	44 (39.29%)
Lymph node	
Positive	30 (26.79%)
Negative	82 (73.21%)

Hormone receptor analysis showed ER positivity in 65.19% of cases, PR positivity in 71.43%, and HER2 positivity in 28.57%. Tumor size ranged from < 2 cm in 5.36% of cases to > 5.0 cm in 39.29% of cases, with the majority (55.36%) between 2.1–5.0 cm. Lymph node involvement was observed in 26.79% of cases. The complete clinicopathological association analyses are presented in Table S3.

## 4. Discussion

This study investigated the association between two biologically relevant SNPs (*ESR1* rs2234693 [T > C] and *PIK3CA* rs6443624 [C > A]) and BC susceptibility in a Bangladeshi cohort. These SNPs were selected due to their functional relevance to key signaling pathways frequently implicated in breast tumorigenesis: estrogen signaling and the PI3K/AKT/mTOR pathway [28–30]. Despite limited prior data from South Asian populations, these loci are widely studied in other regions, and their pathway‐level importance provided a strong rationale for focused analysis.

Our results show that the CT genotype at *ESR1* rs2234693 is significantly associated with a reduced risk of BC across additive, dominant, and over‐dominant genetic models. As *ESR1* encodes ER*α*, a transcription factor regulating genes involved in proliferation and apoptosis in estrogen‐sensitive tissues, variants in its regulatory regions may alter receptor expression [31–34]. Although rs2234693 is intronic, prior evidence suggests it may influence transcription factor binding or chromatin structure, thereby modulating ER*α* activity [28, 35].

The protective effect of CT heterozygosity may represent a balanced modulation of estrogen signaling which is enough to sustain physiological function without tipping toward the hyperproliferative signaling often implicated in hormone‐driven cancers [36–38]. This hypothesis aligns with findings from Deroo and Korach [39], though other studies have reported no association [40], reflecting interpopulation variability and possible gene–environment interactions.

In contrast, the CA genotype at *PIK3CA* rs6443624 was associated with a significantly increased risk of BC, particularly under additive and dominant models. As *PIK3CA* encodes the p110*α* catalytic subunit of PI3K, which initiates downstream AKT/mTOR signaling, its dysregulation is a well‐established driver of tumorigenesis [21, 41, 42]. Although rs6443624 is located in a noncoding region, intronic SNPs may impact gene expression, mRNA stability, or alternative splicing [43–45].

Our results support previous findings suggesting that *PIK3CA* alterations contribute to BC, though not all studies have shown significant associations for rs6443624. For example, Ulu et al. [27] found no such correlation in a Turkish population, pointing again to population‐level differences in linkage disequilibrium patterns or environmental modifiers. In the present study, the heterozygous CA genotype consistently exhibited an association with increased breast cancer risk under the additive, dominant, and over‐dominant genetic models, indicating that heterozygosity at this locus may influence genetic susceptibility. Nevertheless, the rarity of the CC genotype limited the statistical power of the Additive 2 and recessive models, and these findings should therefore be interpreted with caution until validated in larger cohorts.

To further investigate the potential clinical relevance of the investigated polymorphisms, genotype–clinicopathological association analyses were performed. No significant associations were observed between either *ESR1* rs2234693 or *PIK3CA* rs6443624 and ER status, PR status, HER2 status, tumor grade, or tumor size. Notably, *ESR1* rs2234693 showed a significant association with lymph node involvement, suggesting a possible relationship with disease progression. However, this finding should be interpreted cautiously because of the limited sample size and requires confirmation in larger independent cohorts.

Although the present findings support the potential contribution of *ESR1* rs2234693 and *PIK3CA* rs6443624 to breast cancer susceptibility, the biological mechanisms underlying these associations remain to be elucidated. Because both variants are located within intronic regions, they may influence gene regulation through effects on transcription factor binding, chromatin organization, alternative splicing, or other regulatory mechanisms affecting gene expression. Future functional investigations integrating molecular experiments with bioinformatic analyses will be essential to clarify the biological significance of these variants and their potential utility in breast cancer risk assessment.

The contrasting associations observed for these two SNPs reflect the complexity of BC genetics. Both *ESR1* and *PIK3CA* are components of interconnected signaling networks: ER*α* can interact with the PI3K/AKT pathway through nongenomic signaling, creating regulatory feedback loops between hormone signaling and cellular growth pathways [28, 29]. Dysregulation at either node may disrupt this balance, promoting tumorigenesis through different mechanisms.

The subgroup analyses further demonstrated that the observed genetic associations were generally consistent after stratification by age and menopausal status. The protective association of *ESR1* rs2234693 appeared to be more pronounced among younger and premenopausal women, whereas the association between *PIK3CA* rs6443624 and breast cancer susceptibility remained evident across both age and menopausal subgroups. These findings suggest that the contribution of *ESR1* polymorphism may be influenced by hormonal status, whereas the effect of *PIK3CA* appears to be relatively independent of these demographic factors. Nevertheless, these subgroup analyses should be interpreted cautiously because of the limited sample size within individual strata.

From a translational perspective, these SNPs may hold promise as potential genetic susceptibility markers for BC risk stratification, but this potential requires validation in larger, multiethnic cohorts supported by functional studies. However, functional validation and replication in larger, more diverse cohorts are essential before considering clinical applications. Integration with data on hormonal exposure, reproductive history, and lifestyle would offer more comprehensive risk modeling.

Our demographic analysis showed no significant differences between BC patients and HC in terms of age, BMI, occupation, or number of children. This suggests that the groups were well matched, reducing the risk of bias from these variables. The lack of association between BMI and BC risk aligns with earlier findings from Bangladesh [46, 47], although international studies (particularly in postmenopausal women) have reported a stronger link, underscoring the need for further exploration [48]. Similarly, the comparable number of children in both groups indicates that reproductive history may not substantially influence BC risk in this population. Other factors, including education level, socioeconomic class, residential status, and menopausal status, also showed no significant differences, further reducing the likelihood that the observed genetic associations were substantially influenced by demographic confounding.

Histopathological patterns in our study were consistent with global trends. Invasive ductal carcinoma was the predominant subtype [49, 50], and a large proportion of tumors were hormone receptor positive (ER and PR), which is encouraging given their responsiveness to hormone‐based therapies and generally favorable prognosis [51, 52]. Approximately 29% of patients were HER2‐positive, a rate comparable to reports from other Asian populations, suggesting that a notable subset could benefit from HER2‐targeted treatments [53, 54]. Tumor size analysis revealed that most cancers were detected at medium sizes (2.1–5.0 cm), although nearly 40% were larger than 5 cm at diagnosis, highlighting the continued need for earlier detection and improved screening practices [55]. However, tumor size was not significantly associated with either *ESR1* rs2234693 or *PIK3CA* rs6443624 genotypes. Lymph node involvement was present in about one‐quarter of patients. Although not the majority, lymph node positivity remains a critical factor in staging, treatment planning, and outcome prediction [56]. Interestingly, only *ESR1* rs2234693 showed a significant association with lymph node involvement.

### 4.1. Limitations

This study has several limitations. First, although the overall sample size provided adequate statistical power for the primary associations investigated, the relatively small number of participants limited the analysis of rare genotypes, particularly *ESR1* TT and *PIK3CA* CC, resulting in reduced precision for some genetic models. Second, the study was conducted at a single center and restricted to Bangladeshi women, which may limit the generalizability of the findings to other populations. Third, detailed information regarding ethnic subgroup affiliation was not collected; therefore, the potential influence of population stratification and genetic heterogeneity among minority ethnic groups within Bangladesh could not be evaluated. Fourth, lifestyle and environmental risk factors (smoking, diet, breastfeeding history, and contraceptive use, etc.) were not collected, which may confound genetic associations. Finally, functional assays were not performed to validate the biological impact of the studied SNPs. Consequently, the findings demonstrate statistical associations rather than causal relationships. These limitations highlight the need for larger, multicenter studies involving ethnically diverse populations, comprehensive environmental and clinical data, and functional investigations to validate and extend the present findings.

## 5. Conclusion

The present study provides preliminary evidence that *ESR1* (rs2234693) and *PIK3CA* (rs6443624) polymorphisms may be associated with BC susceptibility in Bangladeshi women. The CT genotype of *ESR1* rs2234693 was associated with a protective effect, reducing the risk of BC, as demonstrated across multiple genetic models. In contrast, the CA genotype of *PIK3CA* rs6443624 is associated with a notably increased risk, highlighting its potential role as a genetic risk factor. These findings underscore the importance of genetic variations in BC risk and may contribute to risk prediction models for genetic screening and personalized risk assessment. However, further studies with larger, multiethnic cohorts and detailed environmental and lifestyle data are needed to validate these associations and to better understand the molecular mechanisms through which these polymorphisms influence BC development.

**Nomenclature**
aOR: adjusted odds ratio
BC: breast cancer
CI: confidence interval
ESR1: estrogen receptor 1
HC: healthy controls
HWE: Hardy–Weinberg equilibrium.
PCR‐RFLP: polymerase chain reaction‐restriction fragment length polymorphism
PIK3CA: phosphatidylinositol‐4,5‐bisphosphate 3‐kinase catalytic subunit alpha
SNPs: single nucleotide polymorphisms
X^2^
: chi‐square value

## Author Contributions

Mahim Hasan Chowdhury: methodology, data curation, investigation, writing—original draft. Faria Billal Shaolin, Nishat Tabassum Khusbu, Upama Gope Puja: visualization, validation, resources, formal analysis. Md. Sharif Hossain: formal analysis, data curation, writing—original draft. Md. Robiul Islam, Akash Majumder, Shah Musallin Hassan: data curation, formal analysis, validation, writing—original draft. Mohiuddin Ahmed Bhuiyan: formal analysis, data curation. Md. Aminul Haque: conceptualization, funding acquisition, methodology, supervision, resources, project administration, writing—review and editing.

## Funding

This work was supported by the Rufaida BioMeds, RBM20230307; and the BRAC University.

## Disclosure

The study was approved by the Institutional Review Board (IRB) of BRAC University (Approval No. BRACUIRB120220005).

## Conflicts of Interest

The authors declare no conflicts of interest.

## Supporting information


**Supporting Information** Additional supporting information can be found online in the Supporting Information section. Table S1: Age‐stratified association of *ESR1* rs2234693 and *PIK3CA* rs6443624 polymorphisms with breast cancer risk (dominant model). Table S2: Menopausal status‐stratified association of *ESR1* rs2234693 and *PIK3CA* rs6443624 polymorphisms with breast cancer risk (dominant model). Table S3: Association of *ESR1* rs2234693 and *PIK3CA* rs6443624 polymorphisms with clinicopathological characteristics among breast cancer patients (*n* = 112).

## Data Availability

The data that support the findings of this study are openly available in Figshare at 10.6084/m9.figshare.32898884.
