# Supplementary material for: ESR1 and PIK3CA Polymorphisms as Potential Genetic Susceptibility Markers for Breast Cancer Risk in Bangladeshi Women: A Case‐Control Study
Source: Hum Mutat. 2026 Jul 30;2026:3651302. doi: 10.1155/humu/3651302 (PMC13424455; doi:10.1155/humu/3651302)
Supplement: Supplementary file 1 — Supporting Information Additional supporting information can be found online in the Supporting Information section. Table S1: Age‐stratified association of ESR1 rs2234693 and PIK3CA rs6443624 polymorphisms with breast cancer risk (dominant model). Table S2: Menopausal status‐stratified association of ESR1 rs2234693 and PIK3CA rs6443624 polymorphisms with breast cancer risk (dominant model). Table S3: Association of ESR1 rs2234693 and PIK3CA rs6443624 polymorphisms with clinicopathological characteristics among breast cancer patients (n = 112). [file HUMU-2026-3651302-s001.docx]

**Supplementary Table S1.** Age-stratified association of *ESR1* rs2234693 and *PIK3CA* rs6443624 polymorphisms with breast cancer risk (Dominant model)

| **SNP** | **Age Group** | **Genetic Model** | **OR (95% CI)** | ***P*-value** |
| --- | --- | --- | --- | --- |
| ESR1 rs2234693 | ≤50 years | CT+TT vs. CC | 0.222 (0.102–0.480) | <0.001 |
|  | >50 years | CT+TT vs. CC | 0.519 (0.234–1.147) | 0.105 |
| PIK3CA rs6443624 | ≤50 years | CA+CC vs. AA | 4.693 (2.185–10.079) | <0.001 |
|  | >50 years | CA+CC vs. AA | 3.321 (1.513–7.285) | 0.003 |
| **Note:** Odds ratios (ORs) and 95% confidence intervals (CIs) were estimated using logistic regression within each age stratum. | | | | |

**Supplementary Table S2.** Menopausal status-stratified association of *ESR1* rs2234693 and *PIK3CA* rs6443624 polymorphisms with breast cancer risk (Dominant model)

| **SNP** | **Menopausal Status** | **Genetic Model** | **OR (95% CI)** | ***P*-value** |
| --- | --- | --- | --- | --- |
| ESR1 rs2234693 | Premenopausal | CT+TT vs. CC | 0.188 (0.078–0.456) | <0.001 |
|  | Postmenopausal | CT+TT vs. CC | 0.510 (0.251–1.038) | 0.063 |
| PIK3CA rs6443624 | Premenopausal | CA+CC vs. AA | 4.810 (2.058–11.242) | <0.001 |
|  | Postmenopausal | CA+CC vs. AA | 3.434 (1.680–7.020) | <0.001 |
| **Note:** Odds ratios (ORs) and 95% confidence intervals (CIs) were estimated using logistic regression within each menopausal subgroup. | | | | |

*Stratified analyses were performed under the dominant genetic model because of the low frequency of the rare homozygous genotypes within individual strata, which limited the stability of estimates under additive and recessive models.

**Supplementary Table S3.** Association of ESR1 rs2234693 and PIK3CA rs6443624 polymorphisms with clinicopathological characteristics among breast cancer patients (n = 112).

| **Clinicopathological characteristic** | **Category** | **ESR1 rs2234693**  **CC** | **ESR1 rs2234693**  **CT+TT** | ***P*-value** | **PIK3CA rs6443624**  **AA** | **PIK3CA rs6443624**  **CA+CC** | ***P*-value** |
| --- | --- | --- | --- | --- | --- | --- | --- |
| **ER status** | Positive | 54 | 19 | 1.000 | 21 | 52 | 1.000 |
|  | Negative | 29 | 10 |  | 11 | 28 |  |
| **PR status** | Positive | 61 | 19 | 0.562 | 27 | 53 | 0.092 |
|  | Negative | 22 | 10 |  | 5 | 27 |  |
| **HER2 status** | Positive | 22 | 10 | 0.562 | 8 | 24 | 0.766 |
|  | Negative | 61 | 19 |  | 24 | 56 |  |
| **Tumor grade** | I | 41 | 11 |  | 15 | 37 |  |
|  | II | 31 | 11 | 0.334 | 15 | 27 | 0.154 |
|  | III | 11 | 7 |  | 2 | 16 |  |
| **Lymph node involvement** | Positive | 27 | 3 | **0.038** | 9 | 21 | 1.000 |
|  | Negative | 56 | 26 |  | 23 | 59 |  |
| **Tumor size** | < 2 cm | 5 | 1 |  | 3 | 3 |  |
|  | 2.1–5.0 cm | 45 | 17 | 0.835 | 16 | 46 | 0.449 |
|  | > 5.0 cm | 33 | 11 |  | 13 | 31 |  |
| **Note:** Data are presented as the number of patients in each genotype category. Associations between genotypes and clinicopathological characteristics were evaluated using Pearson's Chi-square test under the dominant genetic model (ESR1: CC vs. CT+TT; PIK3CA: AA vs. CA+CC). A two-sided *P* < 0.05 was considered statistically significant. Statistically significant results are shown in **bold**. | | | | | | | |
